# Scalp Lipofibromatosis – A case report on comprehensive management in a two-year-old child from Pakistan

**DOI:** 10.12669/pjms.40.12(PINS).11034

**Published:** 2024-12

**Authors:** Nasruddin Ansari, Aleena Shahbaz, Muhammad Tahir Khan, Zubair Ahmad Khan

**Affiliations:** 1 Dr. Nasruddin Ansari, MBBS Postgraduate Resident, Department of Neurosurgery Unit III, Punjab Institute of Neurosciences, Lahore, Pakistan; 2 Dr. Aleena Shahbaz, MBBS Postgraduate Resident, Department of Neurosurgery Unit III, Punjab Institute of Neurosciences, Lahore, Pakistan.; 3 Dr. Muhammad Tahir Khan, MBBS Postgraduate Resident, Department of Radiology, Punjab Institute of Neurosciences, Lahore, Pakistan.; 4 Dr. Zubair Ahmad Khan, MBBS, FCPS Neurosurgery Consultant Neurosurgeon, Department of Neurosurgery Unit III, Punjab Institute of Neurosciences, Lahore, Pakistan.

**Keywords:** Scalp, Solitary Fibrous Tumors, Magnetic Resonance Imaging

## Abstract

Lipofibromatosis is a rare benign soft tissue tumor that primarily affects children. There is limited cytological description and management of this rare condition in the literature which leads to misdiagnosis. The two years old patient first presented with a big, non-tender swelling on the scalp’s right temporoparietal area. Despite receiving chemotherapy for misdiagnosis of small round cell carcinoma on first biopsy, the tumor continued to grow, requiring surgical removal. The histopathology report after surgical excision validated the diagnosis of lipofibromatosis. The cosmetic care of the wound was taken by plastic surgery team. The fact that this case was successfully treated shows how crucial it is to treat rare pediatric cancers with a multidisciplinary strategy that incorporates surgical precision, imaging, histopathology and aesthetic considerations. This strategy highlighted the need of preserving the patient’s quality of life by cosmetic reconstruction in addition to facilitating efficient tumor removal. Emphasizing thorough diagnosis and interdisciplinary management, this case adds important context to the little existing research on lipofibromatosis.

## INTRODUCTION

Childhood lipofibromatosis is a slow-growing tumor made of fatty tissue that is separated by septa around the perimysium.^1^ With a male to female ratio of 2.7:1, it is extremely rare and often affects the distal extremities first, then the trunk, head, and neck. If treatment is insufficient, the recurrence rate is extremely high, ranging from 33% to 72%.^2^ Histopathology is still the primary method of diagnosis in lipofibromatosis due to the condition’s ambiguous presentation and imaging results.^3^ When a tumor gets bigger over time, surgical excision is advised. This is the first case report from Pakistan with radiological features, histopathology, and management of a two years old child with a large right scalp lipofibromatosis.

## CASE PRESENTATION

**Fig.1:**
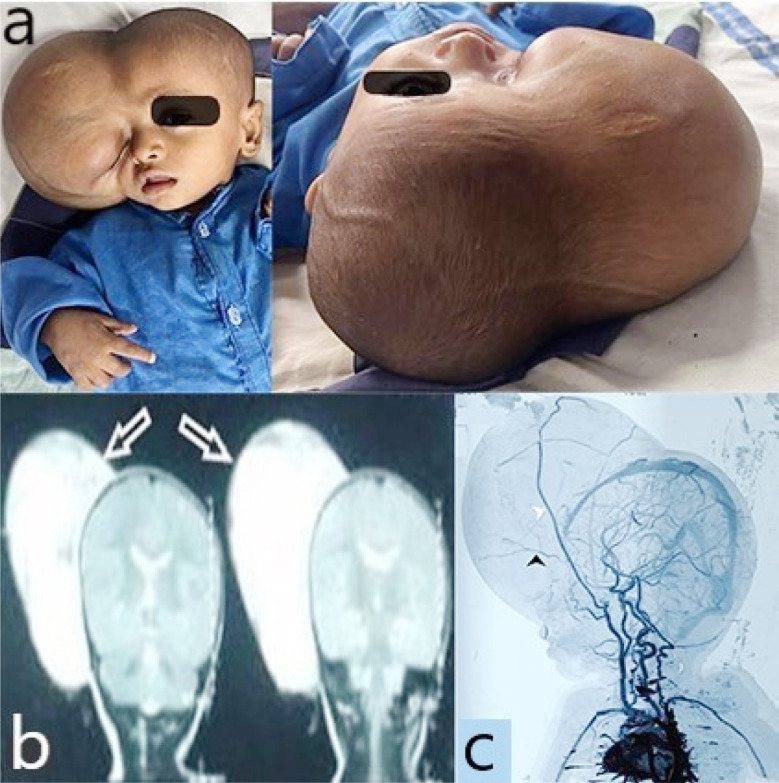
(a) Pictures taken before the surgery, depicting a massive scalp mass. (b) Coronal T2W images showing a large hyperintense fatty lesion (black arrows). (c) CTA brain sagittal image showing the arterial supply of the lesion from superficial temporal artery (white arrowhead) and ophthalmic artery (black arrowhead).

A two years old child was brought by his parents to Punjab Institute of Neurosciences, Lahore after referral from another public sector hospital in September, 2022 with complaints of gradually increasing painless swelling across his right temporoparietal region since birth. On examination the patient was opening eye spontaneously, obeying commands with good cry, and had bilaterally equal and reactive pupils. There was a large swelling of about 15 × 12 cm in size, at right temporoparietal region which was non tender, free of skin overlaying it, and affixed to the underlying structure (Fig.1a). The local temperature was not elevated with regular skin covering and there was no thrill on auscultation. The patient’s vital signs were stable. The child development was age appropriate and had no any systemic abnormality or syndromic affiliation. Based on history and clinical exam lipoma, sebaceous cyst, hamartoma and neurofibroma were kept as differentials. Base line investigation including complete blood count, coagulation profile, renal function tests, liver function test, serum electrolytes and chest X-ray were all within normal limits.

An extracalvarial tumor of 15 x 3 x 7 cm was seen on the brain MRI; there was no intracranial or intraorbital extension (Fig.1b). Brain CT angiography revealed feeders from the ophthalmic and superficial temporal arteries (Fig.1c). Neuroblastoma, infantile fibrosarcoma, tiny round cell sarcoma, and malignant round blue cell tumor were the differential diagnoses.

### Ethics approval and consent to participate:

The parents of patient gave written informed consent for the publication of this case and the necessary radiological pictures. Written informed consent was obtained. This research follows the most recent version of the Helsinki Declaration.

**Fig.2:**
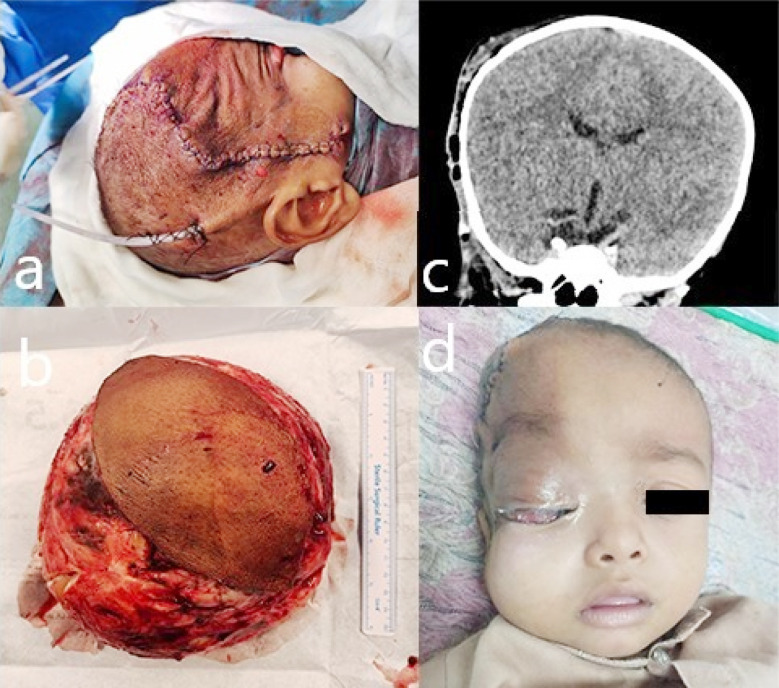
(a) Picture taken at OT table after skin closure and drain insertion. (b) A large tumor after resection. (c) Post-operative CT head coronal view showing edematous changes along the right temporoparietal region. (d) After 3^rd^ post-operative day, periorbital edematous changes are visible.

At birth, the patient’s swelling measured 1 x 1 cm, but it gradually became larger. Five cycles of doxorubicin and ifosfamide were tried without success after a biopsy at another hospital revealed small round cell carcinoma, which resulted in substantial growth. As such, the patient was referred to our institute for further management. After thorough evaluation of the patient surgery was planned. A subcutaneous plane dissection was performed using an elliptical incision. The tumor was excised in its entirety, and the superficial temporal artery was located and ligated. Plastic surgeons preserved the aesthetic closure while excising the excess skin (Fig.2a). The extracalvarial lesion at the temporoparietal region was found to be soft to firm, white, cystic heterogeneous, and fairly vascular throughout the operation (Figure 2b). The intra-operative and post-operative hospital stay was uneventful. The immediate post-operative CT scan of the brain showed no residual tumor (Fig.2c). The subcutaneous drain was removed on 2^nd^ post-operative day and the patient was discharged on 3^rd^ post-operative day (Figure 2d). The specimen was sent to be examined histopathologically. Small, randomly organized round to oval cells were found in the histopathology sections. These cells were deposited in a myxoid background and had negative immunohistochemistry stains (SMA, CD34, MUC4, SOX10, B-catenin, Desmin, S100), which suggested that the diagnosis was infantile lipofibromatosis. The patient followed up on 7^th^ post-operative day had healthy wound healing and stitches were removed. After 6^th^ month and one year of follow up there was no residual mass or swelling and had healthy scar. Currently the baby is active and playful.

## DISCUSSION

Because lipofibromatosis is a rare benign fibrous tumor of children that can recur, it poses special diagnostic and treatment issues.^4^ This instance of a two years old with a large involvement of the scalp sheds light on the difficulties in identifying and treating these uncommon malignancies.

Our case is unique since it is located on the scalp, contrary to the histological description of lipofibromatosis, which is defined by the infiltration of mature adipocytes among fibrous bands, with a preference for the distal extremities.^5^ The diagnosis of juvenile scalp masses requires rigorous histological evaluation due to the wide range of possible causes, from benign entities like dermoid cysts to malignancies like sarcomas.^6^

Imaging is a crucial but complex part of the lipofibromatosis diagnostic maze. The tumor’s size, connection to surrounding structures, and lack of intracranial extension were all determined by magnetic resonance imaging (MRI) in our case. These details are crucial for surgical planning. Although lipofibromatosis is not pathognomonic, its MRI features—such as its well-defined borders and heterogeneous signal intensity owing to the mixing of fibrous and adipose tissue—provide important hints towards the diagnosis.^7^ Moreover, our patient’s CT angiography revealed the vascular supply of the tumor, which is an essential information to minimize intraoperative blood loss and guarantee accurate tumor excision. Despite these benefits, lipofibromatosis cannot be diagnosed with certainty with imaging alone. This is demonstrated by the differential diagnoses that were taken into consideration in our case, such as neuroblastoma and infantile fibrosarcoma, which have similar imaging characteristics to lipofibromatosis. This emphasizes how imaging should not be used as a stand-alone diagnostic tool, but rather as a supplement to histopathological examination. In order to diagnose complex pediatric malignancies like lipofibromatosis, this dual method emphasizes the symbiotic interaction between imaging and histopathology, ensuring precise diagnosis and personalized care.

In order to reduce the risk of recurrence, surgical excision with well-defined margins is the mainstay of lipofibromatosis management. This idea was carefully implemented in our instance through thorough surgical excision. Boose et al., conducted institutional and literature review of uncommon entity of lipofibromatosis and reported recurrence rate ranging from 33% to 72% and emphasized the need for careful long-term follow-up.^2^ However, after the one-year follow-up, our patient showed no signs of recurrence. This could be due to the surgical technique’s thoroughness and the uncommon involvement of the scalp, which may have different biological behavior.

With the initial diagnosis made at another public sector hospital, the patient was put on chemotherapy. This choice was made in light of the difficulty in diagnosing rare tumors like this. The consideration of chemotherapeutic intervention in our situation highlights the importance of obtaining a clear diagnosis prior to beginning treatment, even though it is not a usual method for lipofibromatosis. The tumor’s continued growth in spite of chemotherapy confirms that lipofibromatosis is resistant to these kinds of treatments, which is consistent with research from Butel et al., who found that chemotherapy had a limited effect on these tumors.^8^

Furthermore, the surgical management emphasized the need of interdisciplinary cooperation, with plastic surgery playing a crucial role in aesthetic reconstruction—a factor that is especially important in juvenile situations for both functional and psychological reasons.^9^ The favorable cosmetic result in our instance highlights the significance of including reconstructive factors into the surgical strategy, guaranteeing not only the tumor’s eradication but also the patient’s quality of life being preserved.

## CONCLUSION

In conclusion, the difficulty in identifying and treating such unusual tumors is demonstrated by this case study of childhood lipofibromatosis with uncommon scalp involvement. It underlines the need of surgical excision as a treatment and the critical function that histological examination plays in conjunction with imaging in providing an accurate diagnosis. This case advocates for a multidisciplinary approach to obtain optimal outcomes and improve the quality of life for affected children, adding important knowledge to the limited literature on lipofibromatosis.

### Authors Contribution:

**NA and MTK** conducted literature review, drafted initial document, created images, and amended the final draft.

**MTK** is responsible for the accuracy or integrity of the work.

**AS** oversaw the research and helped with revision.

**ZAK** revised the manuscript and edited images.

The final version of the manuscript was approved by all authors.

## References

[1] Khatri A, Mahajan N, Sengar M, Agarwal A (2020). Lipofibromatosis:A Rare Diagnosis on Fine Needle Aspiration Cytology. Turkish J Pathol.

[2] Boose MD, Chikwava KR, Dormans JP, Chauvin NA, Jen M (2014). Lipofibromatosis:an institutional and literature review of an uncommon entity. Pediatr Dermatol.

[3] Lam YL, Ho WY, Yau R, Lee VWK, Shek TWH (2019). Management of thigh lipofibromatosis in a newborn:a case report. Hong Kong Med J.

[4] Zhang Z, Lu Y, Shi C, Chen M, He X, Zhang H (2023). Pediatric dermatofibrosarcoma protuberans:a clinicopathologic and genetic analysis of 66 cases in the largest institution in Southwest China. Front Oncol.

[5] Porrino J, Al-Dasuqi K, Irshaid L, Wang A, Kani K, Haims A (2022). Update of pediatric soft tissue tumors with review of conventional MRI appearance-part 1:tumor-like lesions, adipocytic tumors, fibroblastic and myofibroblastic tumors, and perivascular tumors. Skeletal Radiol.

[6] Beutler T, Currado B, Tovar-Spinoza Z (2020). Skull tumors and scalp lesions. InTextbook of Pediatric Neurosurgery.

[7] Gupta H, Thaker S (2023). Practical Guide for Imaging of Soft Tissue Tumours.

[8] Butel T, Dumont B, Leruste A, Galmiche L, Pierron G, Pannier S (2020). Orbach D. New born and infant soft tissue sarcomas. Rare Sarcomas.

[9] Rock K, Addison O, Gray VL, Henshaw RM, Ward C, Marchese V (2023). Skeletal Muscle Measurements in Pediatric Hematology and Oncology:Essential Components to a Comprehensive Assessment. Children (Basel).

